# Benchmarking Large Language Model Rationality Using Measurement Axioms

**DOI:** 10.21203/rs.3.rs-10119008/v1

**Published:** 2026-07-01

**Authors:** Kiwon Song, James M. Jennings, Clintin P. Davis-Stober

**Affiliations:** 1 Department of Psychological Sciences, University of Missouri; 2 University of Missouri Institute for Data Science and Informatics

**Keywords:** large language models, AI decision-making, transitivity of preference, measurement theory, utility theory

## Abstract

While Large language models (LLMs) are increasingly deployed as decision-makers, current evaluation practice emphasizes benchmark accuracy rather than the structural properties that make decisions coherent and interpretable. We introduce a measurement-theoretic framework for evaluating AI decision-making using formal axioms derived from utility theory, focusing on transitivity of preference. When transitivity is violated over a choice set, alternatives can no longer be coherently ordered as globally better or worse, undermining the interpretability of model preferences and complicating alignment with human values. We apply our framework across 20 distinct LLMs, systematically varying question format, generation temperature, and contextual memory, using classic choice paradigms previously shown to induce intransitive preferences in humans. We find substantial and systematic transitivity violations in many models, although some patterns partially mirror known features of human decision behavior. Measurement axioms thus offer a principled, complementary foundation for evaluating the rationality and coherence of AI systems beyond benchmark accuracy.

## 1 Introduction

Large language models (LLMs) now occupy decision-making roles across nearly every sector of modern life (Rabanser et al., 2026). They inform credit risk assessments (Goel et al., 2023), assist in patient diagnoses (Jaiswal et al., 2020), and shape policy recommendations across government and education (Kuziemski and Misuraca, 2020; Chen et al., 2020). As these systems increasingly make choices rather than merely produce text, the standard of evaluation must move with them. Getting the right answer some of the time is no longer sufficient; what matters is whether the process behind those answers is consistent and coherent.

Current evaluations of LLMs have largely focused on whether models produce correct, unbiased, and factually grounded outputs (Hendrycks et al., 2021; Liang et al., 2022; Gao et al., 2021). While such work has revealed important limitations, these approaches become difficult to apply in realistic environments where no clear “ground truth” exists. More fundamentally, benchmark accuracy alone may fail to capture whether model-generated decisions are structurally coherent and internally consistent. This omission is notable because preference relations have long played a foundational role in artificial intelligence, underpinning representations of utility, rational agency, plausibility, and strategic choice (Grossi et al., 2022). Recent work has begun to move toward evaluating these broader aspects of AI decision making by testing LLMs against principles of economic rationality (Chen et al., 2023; Wen, 2025), probing for cognitive biases (Koo et al., 2023; Macmillan-Scott and Musolesi, 2024; Simmons, 2022), evaluating LLMs as behavioral simulators (Liu et al., 2024b), and developing rationality-oriented benchmarks (Raman et al., 2024; Xie et al., 2024). Yet despite these advances, there remains no widely adopted formal evaluative framework for determining whether LLM-generated preferences satisfy the structural conditions required for coherent and interpretable decision making (Salaudeen et al., 2025; Laskar et al., 2024; McIntosh et al., 2025; Feuer et al., 2025).

To address this gap, we take a fundamental measurement perspective grounded in classic utility theory (Luce, 2000; Pedersen et al., 2025). We define a decision-making system to be rational, if, and only if, it admits the existence of a utility function (e.g., Von Neumann and Morgenstern, 1947): a mapping u:𝒞→R over a set of choice alternatives 𝒞, such that for any A,B∈𝒞,A is weakly preferred to B if and only if u(A)≥u(B). This definition connects directly to the concept of computational rationality, which frames optimal AI decision-making as identifying choices with the greatest utility subject to computational cost (Gershman et al., 2015). A necessary condition for the existence of such a utility function is the choice axiom *transitivity of preference*: for any A,B,C∈𝒞, if A is preferred to B and B is preferred to C, then A must be preferred to C. Without transitivity, no coherent utility representation exists, and the resulting preference ordering is vulnerable to systematic exploitation (Anand, 1993). Transitivity serves as a diagnostic tool precisely because it evaluates the structural consistency of the decision making outputs rather than the “correctness” of any particular output. It also provides a testable condition against which the effects of operational manipulations can be evaluated. As (Steyvers and Kumar, 2024) argue, establishing whether an AI’s choices are consistent is also essential for managing human expectations in collaborative settings. Recent work has begun to examine transitivity in LLM contexts, including non-transitive preferences in LLM-as-a-judge frameworks (Xu et al., 2025), the emergence of utility-like structures at scale (Mazeika et al., 2025), and value consistency across question formats (Moore et al., 2024). However, these studies either focus narrowly on evaluative judging, assess structural coherence without systematically varying the conditions under which preferences are elicited, or measure consistency without grounding it in formal choice axioms. We address these issues by providing a methodology for assessing transitivity in AI systems that can be applied broadly, across nearly any choice environment, and is sensitive to various environments that can impact decision making behavior.

To evaluate transitivity with the necessary rigor, we adopt a methodology suited to the non-deterministic nature of LLM outputs (Hassija et al., 2023; von Eschenbach, 2021). Where previous studies evaluating AI rationality have relied on variable prompt-engineering strategies or post-hoc parsing of generated text, we utilize direct log-probability extraction, a measurement technique established in foundational LLM benchmarking (Hendrycks et al., 2021; Gao et al., 2021; Liang et al., 2022). Specifically, we extract the model’s response behavior by calculating the first-token probability between two choices, alongside analyzing the greedy search output to capture the model’s immediate intrinsic choice. Log-probability extraction operates below the stochastic generation layer, recovering raw output probabilities that are deterministic for a given input, yielding a stable and reproducible measure of preference independent of generated text variability.

**Figure 1:**
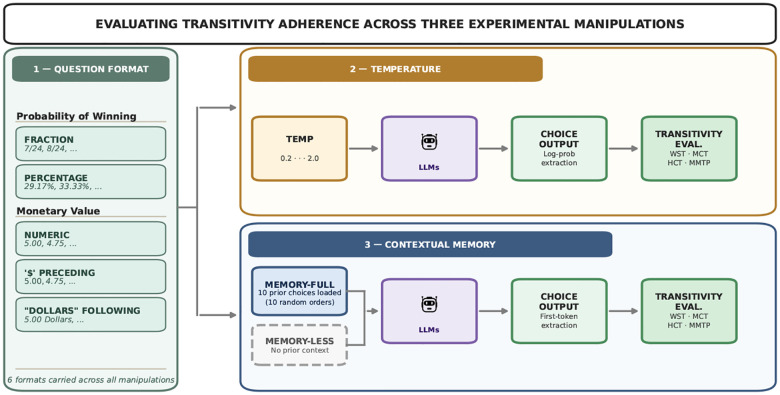
Overview of the experimental design. Six question formats (left) are carried across two manipulations: generation temperature, and contextual memory. Choice outputs are extracted via log-probabilities or first-token extraction and evaluated against four models of transitivity (WST, MCT, HCT, MMTP).

With this evaluative framework in place, we test transitivity across three conditions that shape how an LLM behaves in practice. The first is **the generation temperature**, which controls how deterministic or exploratory a model’s output is (Li et al., 2025), and is among the most commonly adjusted settings in practice (Du et al., 2025), yet no prior work has tested whether increasing it destabilizes the logical structure of preferences rather than simply diversifying outputs. Nested within this manipulation, we also compare the intransitive patterns LLMs produce against the systematic violations documented in human decision-making (Tversky, 1969), offering a direct test of behavioral alignment at the structural level. The second is **contextual memory**, which tests whether providing a model with a record of its prior choices improves or degrades preference consistency. Prior work has documented that as context history grows, agent behavior becomes inconsistent and errors accumulate (Verma, 2026; Xu and Lu, 2025). However, whether this inconsistency extends to the formal structure of preferences is untested. Finally, **question format** tests whether the framing of a choice alters preference structure. While prompt sensitivity is widely known to degrade model accuracy (Sclar et al., 2023; Ngweta et al., 2025), its effect on adherence to formal choice axioms remains unmeasured. By evaluating these specific conditions, we directly test whether they systematically increase or decrease the frequency of intransitive choices. Figure 1 provides an overview of how these three manipulations are structured within the experimental pipeline.

Together, these manipulations demonstrate the broad utility of the transitivity axiom as a benchmarking tool for AI decision-making. Rather than measuring what a model gets right, transitivity testing reveals whether the structure of its decision-making process meets a formal criterion of rationality, providing a more foundational and reproducible standard for output quality than accuracy-based evaluation alone. In doing so, this paper contributes to the theoretical foundation for computational alignment and advances a more rigorous understanding of artificial agent decision-making.

### 1.1 Models of Transitivity

While the transitivity axiom appears, at face value, to be straightforward, several conceptual and technical challenges arise in practice. The core challenge is that transitivity is an algebraic statement; deterministic by definition and does not involve random variables. Applying it directly to systems whose outputs are non-deterministic, whether human, animals, or artificial, requires an additional formal step. This is a well-known challenge in the transitivity literature (Regenwetter et al., 2011, 2021). To address this, various probabilistic models of transitivity have been developed over the past 70 years, each maintaining connections to established choice theories - see e.g., (Davis-Stober et al., 2017). These models are defined probabilistically, considering the probability with which A is chosen over B. For human subjects, estimating these probabilities requires repeated trials and statistical inference. LLMs, however, can provide them directly; for any binary choice, the model’s first-token log-probabilities yield the exact probability of each token being selected, making these probabilistic models of transitivity a natural test of structural consistency for these systems.

As we demonstrate later, the probability an LLM assigns to one alternative over another shifts with the conditions under which the choice is elicited, including temperature, format, and contextual memory. This variability is precisely what the probabilistic models described below are designed to evaluate. To be clear, the goal is not to recover a “cognitive process” underlying LLM responses. Rather, we take a measurement perspective: support for, or against, a given probabilistic model of transitivity informs the degree of consistency in the LLM’s output, specifically what class of choice functions can reasonably describe the behavior being generated.

Building on the QTest framework for evaluating human responses (Regenwetter et al., 2014), we consider four probabilistic models of transitive choice: weak stochastic transitivity (WST), medium-certain transitivity (MCT), high-certain transitivity (HCT), and the mixture model of transitive preference (MMTP). Each model differs in the strictness of the conditions it imposes on choice probabilities, and together they provide a graded framework for evaluating the degree to which an LLM’s preferences are structurally consistent.

#### Weak Stochastic Transitivity (Davidson and Marschak, 1959).

Let $P_{AB}$ denote the probability of alternative A being selected over alternative B, where A and B are members of a set 𝒞 of choice alternatives. WST holds if, and only if,

(1) $$P_{AB}\geq \frac{1}{2}\wedge P_{BC}\geq \frac{1}{2}\Rightarrow P_{AC}\geq \frac{1}{2},\;(\forall A,B,C\in 𝒞),$$

where “∧” denotes conjunction. WST is a necessary and sufficient condition for the existence of a utility function u such that

$$u(A)\geq u(B)\Leftrightarrow P_{AB}\geq \frac{1}{2},\;(\forall A,B\in 𝒞).$$

This condition defines what is referred to as a weak utility model (Roberts, 1979). As the least restrictive of the four models considered, WST establishes the foundational criterion for transitive consistency, with MCT and HCT imposing progressively more demanding conditions on the choice probability structure.

#### Medium-Certain Transitivity.

MCT proposes that an agent’s preferences should be maintained at a moderate but non-trivial level of certainty, stricter than chance but less demanding than near-certain consistency. Formally,

(2) $$P_{AB}\geq 0.75\wedge P_{BC}\geq 0.75\Rightarrow P_{AC}\geq 0.75,(\forall A,B,C\in 𝒞).$$

#### High-Certain Transitivity.

HCT is a principle of logical consistency designed for high-certainty agents such as LLMs. It proposes that for an agent’s preferences to be considered consistent, that consistency must be maintained at a high and non-trivial level of probability. Formally,

(3) $$P_{AB}\geq 0.9\wedge P_{BC}\geq 0.9\Rightarrow P_{AC}\geq 0.9,(\forall A,B,C\in 𝒞).$$

The formulation of MCT and HCT is motivated by a fundamental difference between human and AI decision-making. The 0.5 threshold of WST was designed for agents whose choices are subject to perceptual noise, guessing, and fatigue (Tversky, 1969; Regenwetter et al., 2011). LLMs, by contrast, produce deterministic probability computations for a given input, and a probability of 0.75 or 0.9 carries meaningfully less ambiguity than one at chance. By establishing these stricter criteria, MCT and HCT provide a more demanding test of logical consistency that is better suited to the architectural properties of the systems being evaluated.

#### Mixture Model of Transitive Preference.

The mixture model of transitive preference (MMTP), also referred to as the linear ordering polytope (Grötschel et al., 1985) or the random preference model of transitive choice (Loomes and Sugden, 1995), allows for an individual, whether human, animal, or LLM, to generate stochastic choice responses by randomly sampling over transitive, deterministic preferences. Following the notion used by (Cavagnaro and Davis-Stober, 2014), let 𝒯 be the set of all complete, asymmetric, transitive binary relations on 𝒞. An individual satisfies MMTP if, and only if, there exists a discrete probability distribution θ over 𝒯 such that

$$P_{AB}=\sum_{T\in 𝒯|(A,B)\in T}\theta (T),$$

for all A,B∈𝒞, where θ(T) is the probability that the individual is in transitive state T, and (A,B)∈T denotes that A is ranked ahead of (preferred to) B in the relation T. For choice sets 𝒞 containing up to five distinct elements, MMTP is completely described by the following three inequalities:

(4) $$P_{AB}+P_{BC}-P_{AC}\leq 1\;(\forall A,B,C\in 𝒞),$$

(5) $$P_{AB}\geq 0\;(\forall A,B\in 𝒞),$$

(6) $$P_{AB}+P_{BA}=1\;(\forall A,B\in 𝒞).$$

See (Regenwetter et al., 2021) for a recent discussion of obtaining minimal descriptions of MMTP for |𝒞|>5. It is important to note that a collection of choice probabilities, ${\left(P_{AB}\right)}_{A,B\in 𝒞,A\neq B}$, satisfying WST (resp. MMTP) does not imply that it satisfies MMTP (resp. WST). See (Davis-Stober et al., 2017) for a discussion and review of how WST and MMTP are necessary conditions for various classes of decision theories.

### 1.2 AI Models considered

This study evaluated 20 distinct LLMs from prominent developers, including Meta, Alibaba (Qwen), MistralAI, and Google. The selected models vary significantly in parameter count, architecture, and training methodology, allowing for a broad examination of transitivity across the current landscape of available models.

A primary distinction among the selected models is their training stage: **Base models** versus **Instruct (Chat) models.** Base models are foundational networks trained on broad corpora, which often require specific prompting techniques (e.g., few-shot examples) to generate structured outputs. In contrast, Instruct models are fine-tuned versions of these base models, optimized via Supervised Fine-Tuning (SFT) and Reinforcement Learning from Human Feedback (RLHF) to follow user instructions and engage in dialogue. The models also differ by parameter count, the number of variables estimated during training. Generally, a higher parameter count correlates with the ability to capture more complex patterns and reason more abstractly (Goodfellow et al., 2016).

The largest group of models tested belonged to the Meta Llama family, including base and instruct/chat versions of the Llama 2 (7B, 13B, 70B) and Llama 3 (8B, 70B) series (Touvron et al., 2023; Dubey et al., 2024). To assess the impact of fine-tuning advancements, this group also included an official update (Llama-3.3-70B-Instruct) and a community-tuned variant (Centaur-70B) (Meta, 2024; Binz et al., 2025). The Centaur-70B was specifically trained on a curated dataset of psychological tests to improve performance on tasks requiring human-like reasoning.

## 2 Method

To provide broader comparative context, the study also included models from Alibaba’s Qwen series, primarily from the Qwen 3 generation, spanning sizes from 0.6B to 32B parameters (Team, 2024). Both the base and instruct versions of MistralAI’s Mistral-Small-24B were included to test a performance-optimized architecture (Mistral AI Team, 2025). Finally, Google’s Gemma-3-27B-it, an instruction-tuned model derived from Gemini series research, rounded out the selection (Team et al., 2025).

Notable closed-source commercial models such as OpenAI’s GPT-5 or Google’s Gemini Pro were excluded from this study for two reasons. First, there are concerns of reproducibility. Commercial APIs frequently update their backend infrastructure, meaning the same model version may yield different results over time. Second, access to consistent output probabilities. Closed-source models are subject to undisclosed post-training interventions that may override the model’s intrinsic probability assignment, introducing a confound that cannot be accounted for.

A detailed list of all 20 models, including their Hugging Face identifiers and specific version IDs, is provided in Table 2 in the Appendix.

### 2.1 Stimulus Design

The experimental task required each LLM to complete a series of choice trials. In each trial, the model was prompted to select a preferred gamble from a presented pair. These pairs were drawn from five distinct stimulus sets adapted from established human transitivity experiments: three sets from (Tversky, 1969) and two sets from (Cavagnaro and Davis-Stober, 2014). Each stimulus set comprised five gambles, with each gamble defined by a specific monetary value and a corresponding probability of winning. For each set, all permutations of two gambles were generated, resulting in 20 choice trials per set.

To systematically investigate the effect of question format, a well-documented source of performance variation in LLMs, six distinct presentation formats were constructed for each gamble pair. These formats varied the representation of probability and monetary value. The probability of winning was expressed either as a fraction (e.g., 7*/*24) or as a percentage (e.g., 29.16%). The monetary value was presented in three distinct ways: as a numeric value alone (e.g., 5.00), with the word “dollars” appended to the value (e.g., 5.00 dollars), or with the dollar symbol preceding the value (e.g., $5.00).

Each gamble pair and question format was presented across all 20 LLMs. The specific attributes for each gamble set are detailed in Table 1. Examples of the prompts used across all formats are provided in the Appendix A.2.1.

### 2.2 Computational Infrastructure

A custom Python framework was developed to automate the experimental pipeline, including prompt generation, model implementation, and response recording. Models were instantiated for text generation and probability extraction using the Hugging Face transformers library. To accommodate the significant computational requirements of testing 20 distinct LLMs, particularly the 70B parameter variants, the experiment was conducted using high-performance computing (HPC) infrastructure. Detailed specifications of the computing cluster and hardware resources are provided in the Appendix.

### 2.3 Experimental Manipulations

Beyond the question format variations described in the previous section, two additional experimental conditions were systematically varied to evaluate their influence on transitive consistency: generation temperature, which controls the shape of a model’s output distribution, and contextual memory, which determines whether a model has access to a record of its prior choices.

#### 2.3.1 Generation Temperature

Generation temperature controls how deterministic or exploratory a model’s output distribution is, and is among the most commonly adjusted settings in practice (Du et al., 2025). To measure its effect on transitive consistency, we employed a **First-Token Probability** extraction method, which adapts established frameworks for evaluating LLMs on multiple-choice tasks (Santurkar et al., 2023; Scherrer et al., 2023; Kauf et al., 2024). This method directly analyzes the log-probabilities a model assigns to specific candidate tokens, providing a continuous measure of choice probability without requiring the model to generate text.

Temperature governs the shape of the output distribution from which token probabilities are derived. At lower temperatures, the distribution becomes more peaked, concentrating probability mass towards the most likely choice. At higher temperatures, it flattens, spreading probability more evenly across alternatives. It is this shift in distributional shape, rather than stochastic sampling, that the current manipulation captures. To cover the full spectrum of model behavior, the extraction process was repeated under 10 temperature settings ranging from 0.2 to 2.0 in increments of 0.2.

To ensure unambiguous data, models were prompted to select between alternatives ‘1’ and ‘2’. The log-probabilities of the top 5 predicted tokens were then extracted. In preliminary testing and throughout the formal trials, the tokens representing the valid choices (‘1’ or ‘2’) were consistently the two most likely outputs across all models. To isolate the choice probability, the probabilities for these two tokens were normalized to sum to 1.0. The choice probability $P_{ij}$ was defined as the resulting normalized probability at each temperature level.

#### 2.3.2 Contextual Memory

The second manipulation tested whether providing a model with a record of its prior choices influences transitive consistency. Most commercial LLMs operate within a context window, retaining interaction history by passing prior conversations back with each new prompt. For example, Llama 2 architecture supports a 4,096-token limit (Touvron et al., 2023), while Llama 3 expands this to 128,000 tokens (Dubey et al., 2024). Whether this capacity for sequential context stabilizes or destabilizes the structure of preferences is an open question.

Due to computational demands of processing extended context histories, this manipulation was restricted to twelve instruction-tuned models (n=12). This subset comprised Llama 2 7B Base and Chat, Llama 2 13B Base and Chat, and Llama 3 8B Base and Instruct from the Llama family; Qwen 3 0.6B, Qwen 3 4B, and Qwen 3 8B from the Qwen family; and Mistral Small 24B Base and Instruct and Gemma 3 27B Instruct.

Two conditions were implemented. In the **Memory-Less** condition, each of the 20 choice trials within a gamble set was presented independently, with no prior context provided. In the **Memory-Full** condition, the model received a record of its 10 most recent choices before each new trial, allowing prior decisions to inform subsequent ones. Both conditions were run at the default generation temperature of 1.0, keeping the output distribution consistent with standard model behavior and constraining the effect of temperature-driven variations.

The memory condition used the same **First-Token Probability** extraction method as the temperature manipulation. Models were prompted to select between alternatives ‘A’ and ‘B’, the log-probabilities of the top 5 predicted tokens were extracted, and the probabilities for the two valid choice tokens were normalized to sum to 1.0. The resulting choice probability $P_{ij}$ served as the measure of preference for each trial.

To construct the prior context required for the **Memory-Full** condition, a discrete choice label was derived from each trial’s probability distribution. The gamble with the higher normalized probability was recorded as the model’s prior choice. For example, if an LLM assigned A a probability of 0.70 and B of 0.30, the choice that was passed into the next trial’s context was recorded as A. This approach provided an unambiguous discrete record that remained stable across the full sequence of decisions.

To control for potential order effects, the presentation sequence of the 20 choice pairs within each gamble set was randomized. Ten distinct orderings were generated which provides a more robust measure of whether transitive consistency holds under the memory condition, and allow for an assessment of whether the sequence in which prior choices are encountered influences the structure of subsequent decisions. The prompting templates and randomized orderings are detailed in Table 3 in the Appendix.

### 2.4 Data Aggregation

For each experimental condition, binary choices were aggregated by LLM, gamble set, and question format. This initially yielded 20 vectors of choice pairs per gamble set, where each data point represented the count of one alternative being selected over another. To assess positional bias, the permuted pairs (e.g., AB and its reverse BA) were first analyzed separately.^1^ To control for this positional bias, these pairs were then averaged into an Aggregated choice probability. This single metric represents the overall preference for Gamble A over Gamble B, producing a final set of 10 choice vectors per condition, which serve as the default measure for all transitivity analyses reported in the Results.

For all three presentation-ordered vectors, a direct conditional check of transitivity was performed across the 10 possible triads within a gamble set (e.g., ABC, ABD, …, CDE). The analysis verified whether the choice probabilities satisfied the conditions of each of the four models of transitivity (WST, MCT, HCT, MMTP). Based on this verification, a binary score was assigned. If any of the 10 triads within a set failed to meet the specific condition, the LLM for that experimental condition was assigned a score of 0, representing a failure of transitive adherence for that model of transitivity. A score of 1 was assigned if and only if all triads strictly met the conditions, representing full adherence.

For the memory condition, each of the 10 randomized orderings was evaluated independently to each transitivity model. Each orderings results are reported separately in the Results, allowing for an assessment of whether the sequence in which prior choices are presented influences transitivity structure and choice consistency.

## 3 Results

The results are organized according to the three experimental manipulations described in the Method. Section 3.1 reports the effects of generation temperature on choice probability and transitivity adherence, and compares the intransitivity patterns produced by LLMs against those documented in human decision-making. Section 3.2 examines the influence of question format on choice probability and transitivity across the six presentation conditions. Section 3.3 evaluates the effects of contextual memory, comparing the Memory-Less and Memory-Full conditions. Two supplementary analyses are reported in the Appendix, positional bias and it’s effect on adherence, and an examination of whether the model reproduces the same choice when a counterbalanced pair reoccur after varying numbers of intervening choice.

### 3.1 Generation Temperature

#### 3.1.1 Transitivity Adherence

**Figure 2:**
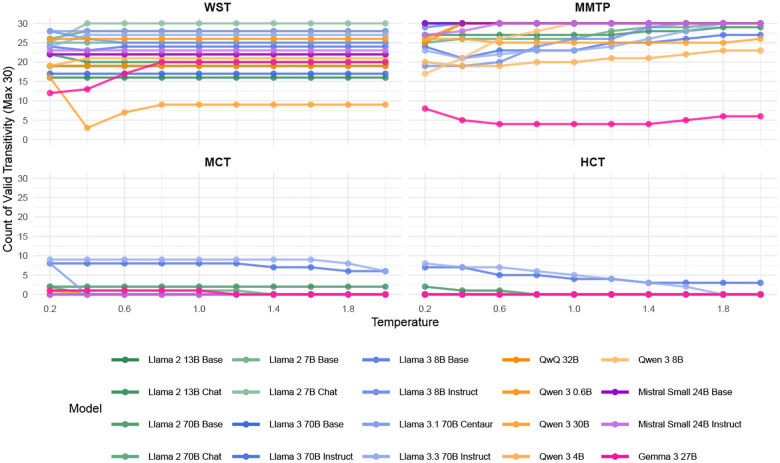
Count of valid transitivity adherence for each LLM across the four transitivity models (WST, MMTP, MCT, HCT) as a function of generation temperature. The maximum count of 30 corresponds to the 30 unique conditions (5 gamble sets × 6 question formats). Values reflect the Aggregated choice probabilities.

Figure 2 presents the count of valid transitivity adherence (out of 30) for each of the 20 LLMs across the four transitivity models as a function of generation temperature.

From the analysis, WST adherence remained stable across all temperature levels with no systematic change as temperature increased. This stability aligns with expectations, given that WST imposes the least restrictive condition, requiring only that choice probabilities exceed 0.5. Adherence varies considerably across models, with counts ranging from 9 to 30 out of 30. Interestingly, instruction-tuned models consistently produced higher WST adherence than their base counterparts (mean of 24.4 vs. 19.9).

MMTP adherence followed a different pattern than WST. Across models, MMTP counts were generally high and increased slightly with temperature, rising from a mean of 25.2 at temperature 0.2 to 28.1 at temperature 2.0. Most models reached full adherence at higher temperatures, though Gemma 3 27B was a notable exception, remaining low across all temperatures (approximately 5 to 6 out of 30). The base vs. instruction-tuned pattern also reversed, where base models produced higher MMTP adherence than their instruction-tuned counterparts (mean of 28.5 vs. 24.9 out of 30).

MCT and HCT adherence was near zero for all models after aggregation. Only Llama 3 70B Instruct (MCT = 8, HCT = 4) and Llama 3.3 70B Instruct (MCT = 9, HCT = 5) maintained non-zero counts, and both declined with temperature. No base model satisfied MCT or HCT in any condition, and both models that did were instruction-tuned 70B variants, suggesting that scale and fine-tuning may both be important factors for adherence to the stricter transitivity conditions.

A parallel analysis using the probabilities before the aggregation produced a different picture, particularly for MCT and HCT, where strict-transitivity adherence was substantially higher before aggregating the two probabilities. Since this comparison speaks directly to positional bias rather than on temperature effects, it is reported in Appendix B.1.

#### 3.1.2 Systematic Intransitivity

Beyond measuring whether LLMs satisfy formal models of transitivity, we examined the structure of their violations: whether the intransitive choices aligned with the patterns documented by (Tversky, 1969) in human decision-makers. Tversky proposed that participants in his experiments followed a **lexicographic semi-order**, a decision rule evaluating attributes sequentially by importance, where a secondary attribute resolves the choice only if the difference in the first attribute falls below a noticeable difference threshold. The rule extends Luce’s semi-order (Luce, 1956) to multiple attributes, and was later given a probabilistic formulation by Davis-Stober (Davis-Stober, 2012). In the context of gambles, if the difference in winning probability (the first attribute) is negligible, the decision defer to the monetary prize (the second attribute); if the probability difference exceeds the threshold, probability alone will dictate the choice.

In our five stimulus sets, all sequence of five gambles the probability of winning increased in equal, small steps while the monetary value decreased correspondingly, which was desgined to expose this rule. We refer to gambles that are one step apart in this sequence as **adjacent pairs**, where the probability of winning difference is small and the lexicographic semiorder predicts choice by monetary value, and to gambles separated by multiple steps as **distant pairs**, where the probability difference is large and the rule predicts choices by probability.

To determine whether LLMs replicate this specific pattern, we analyzed their aggregated choice probabilities (at the default temperature of 1.0) across the five gamble sets (Table 1). Because these sets are structured so that monetary value decreases while the probability of winning increases in alphabetical order, they allow us to classify the responses for each triad (e.g., A,B,C) into distinct intransitive cycles. We defined **Human-aligned intransitivity** as instances where the LLM followed the lexicographic semi-order: preferring the higher-value gamble in adjacent pairs (where the probability difference is small, $P_{AB}\geq 0.5$ and $P_{BC}\geq 0.5$) but reverting to the higher-probability gamble in the distant pair (where the probability difference is large, $P_{AC}<0.5$). Conversely, **Non-Human intransitivity** was defined as the opposite cycle, where the LLM preferred the higher-probability gamble in adjacent pairs but reversed to the higher-value gamble in the distant pair. Ultimately, each LLM yielded 300 triad classifications across the combinations of gamble sets and question formats.

**Figure 3:**
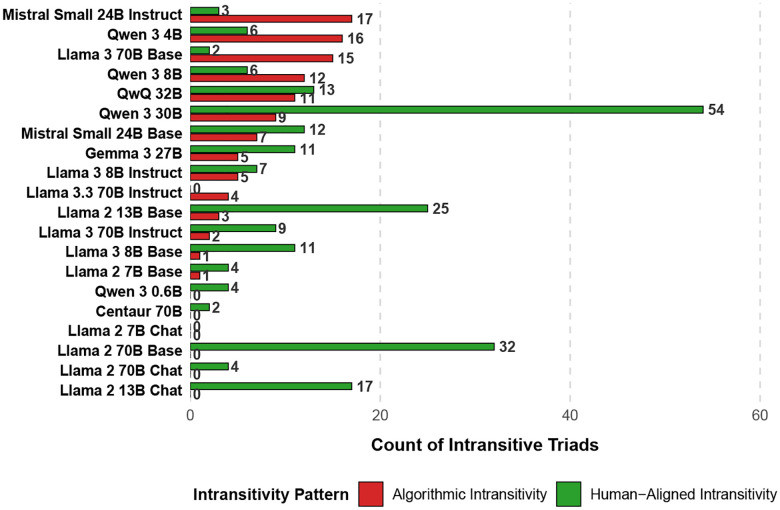
Count of human-aligned and non-human intransitive triads for each LLM using Aggregated choice probabilities at temperature 1.0. Counts are summed across all 5 gamble sets and 6 question formats (10 triad per set, 300 total triads per model).

Figure 3 presents the count of human-aligned and non-human intransitivity triads for each of the 20 LLMs. Across all LLMs, 222 total triads were classified as human-aligned while 108 were not. Of the 20 models, 14 produced more human-aligned intransitive triads, while 5 showed the opposite pattern, and 1 (Llama 2 7B Chat) produced none of either type.

The direction and frequency of intransitivity varied across model families, and results did not reveal a consistent relationship with either parameter count or instruction tuning. Within the Llama 2 Base family, total intransitive triads increased with parameter count (5 at 7B, 28 at 13B, 32 at 70B) and were predominantly human-aligned. However, this did not generalize to other families, for example, Llama 3 70B Base showed the opposite tendency (2 human, 15 non-human). Similarly, instruction tuning lacked a uniform effect — it shifted Llama 3 70B towards human-aligned intransitivity (from 2 to 9 human-aligned triads), but produced the reverse shift in Mistral Small 24B. Among all 20 models, Qwen 3 30B was a notable case, producing 54 human-aligned intransitive triads, substantially more than any other model.

### 3.2 Question Format

**Figure 4:**
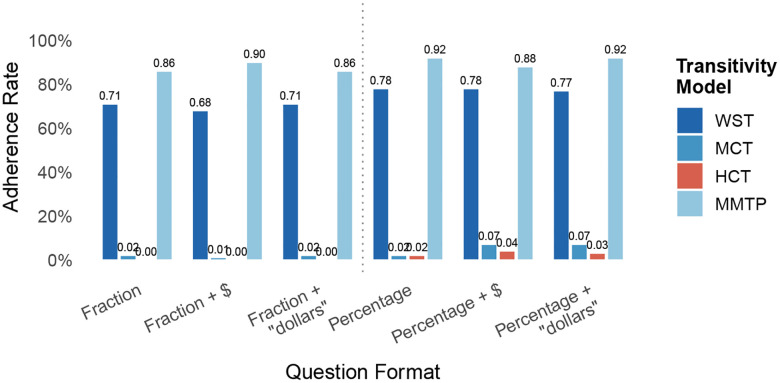
Mean transitivity adherence rate across all 20 LLMs for each of the four transitivity models (WST, MMTP, MCT, HCT) and six question formats. Values reflect Aggregated choice probabilities at temperature 1.0.

The next manipulation examined the influence of question format on transitivity adherence. Figure 4 presents the mean transitivity adherence rate across all 20 LLMs for each of the four transitivity models and six question formats, using Aggregated choice probabilities at temperature 1.0. Across all four transitivity models, question format produced modest differences in adherence.

The variation that emerged was primarily driven by how the probability of winning was presented, rather than the presentation of monetary value. When probability was expressed as a percentage rather than a fraction, transitivity adherence was higher across every transitivity model: WST (0.78 vs. 0.70), MMTP (0.91 vs. 0.87), MCT (0.05 vs. 0.02), and HCT (0.03 vs. 0.00). This effect was most noticeable for HCT, where every non-zero value observed occurred under a percentage format. By contrast, the three monetary value formats (numeric, dollar symbol, and the word “dollars”) produced almost no variation in adherence within any transitivity models (differences range from 0.00 to 0.025 across the four transitivity models).

### 3.3 Contextual Memory

#### 3.3.1 Memory-Less vs Memory-Full

**Figure 5:**
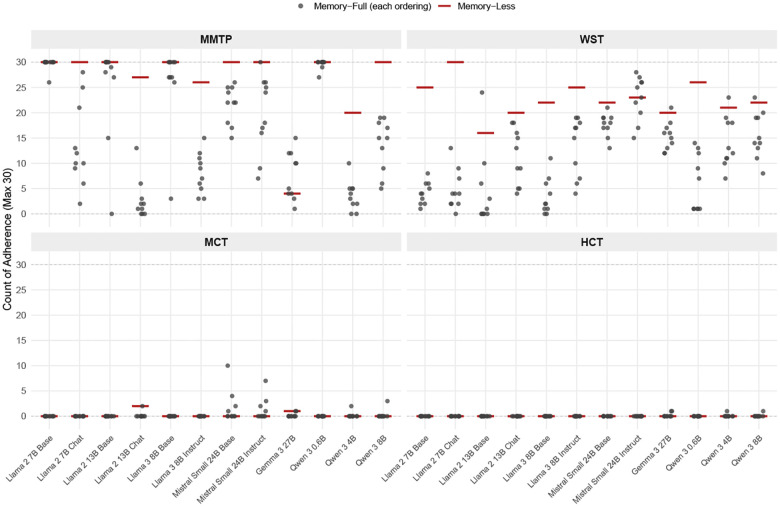
Transitivity adherence (out of 30) by model and transitivity model (MMTP, WST, MCT, HCT). Grey dots represents each of the 10 randomized Memory-Full orderings. Red line for each mode represents the Memory-Less count. Adherence is summed across 5 gamble sets and 6 question formats.

The final manipulation examined whether providing LLMs with a record of their prior choices influenced transitive consistency. Both the Memory-Less and Memory-Full conditions were run at the default generation temperature of 1.0. The manipulation was restricted to the twelve models described in the Method section. Figure 5 presents the count of valid transitivity adherence (out of 30) for each of the 12 LLMs across the four transitivity models, comparing the Memory-Less condition (red line) to the 10 randomized orderings of the Memory-Full condition (grey dots).

The most notable effect of memory was a decrease in adherence for MMTP and WST. Mean Memory-Full adherence, averaged across the 10 orderings, was lower than the Memory-Less count for both MMTP (26.42 → 16.73) and WST (22.67 → 11.06). At the LLM level, 11 of 12 models decreased on MMTP, and all 12 models decreased on WST. The single model that ran against the MMTP direction was **Gemma 3 27B** (4 → 7.60). With this exception noted, the addition of contextual memory decreased adherence to MMTP and WST rather than stabilizing it.

For the stricter MCT and HCT conditions, no comparable shift was observed since adherence was already at floor under Memory-Less. Mean Memory-Less adherence was 0.25 for MCT and 0.00 for HCT, with only **Llama 2 13B Chat** (MCT = 2) and **Gemma 3 27B** (MCT = 1) producing any non-zero adherence. Under Memory-Full, mean adherence remained low (MCT = 0.33; HCT = 0.03). Even with contextual memory of prior choice, LLMs did not lift adherence out of the near-zero range.

Beyond the average shift between conditions, the 10 orderings produced within-model variability in the Memory-Full condition. The mean range across orderings (max − min) was 15.58 for MMTP and 13.17 for WST. The most extreme case was **Llama 2 13B Base**, where MMTP count spanned the full possible range across the 10 orderings (0 to 30). Other models showed similarly wide ranges on MMTP, including **Llama 3 8B Base** (3 to 30), and **Llama 2 7B Chat** (2 to 28). For MCT and HCT, ranges were near zero (means of 2.08 and 0.25). Across the 10 orderings, the same model evaluated under the same memory window can produce quite different counts depending on which ordering is sampled.

These results indicate that contextual memory does not stabilize transitive consistency. For MMTP and WST, memory shifted adherence downward, and within Memory-Full the counts varied significantly across the 10 orderings. For MCT and HCT, neither condition produced more than near-zero adherence, therefore, no shift was identifiable. A further analysis examining whether models reproduced the same choice on counterbalanced pairs is reported in Appendix B.1.1.

## 4 Discussion

This study introduced a formal framework to evaluate the structural consistency of Large Language Models (LLMs), grounded in utility theory, utilizing adherence to stochastic interpretations of the transitivity axiom (WST, MMTP, MCT, and HCT) as a reproducible benchmark for computational rationality. By employing direct first-token probability extraction, we systematically evaluated how fundamental operational parameters, such as generation temperature, contextual memory, and question format, can impact the logical coherence of LLM preferences.

Across 20 different LLMs, the framework’s application suggests that while these models may initially appear to exhibit structured decision-making, their failure to produce consistent preferences across different conditions indicates the absence of a stable, utility representation. For instance, while increasing generation temperature raised MMTP adherence, this improvement is likely a mathematical consequence of compressing choice probabilities toward ‘coin-flip’ guessing (p=.5) for all choice probabilities (a value that is consistent with MMTP) and not a sign that preferences became more coherent. Question format produced a smaller but directionally consistent effect, with adherence shifting based merely on whether probability was written as a fraction or a percentage. Perhaps most interesting, providing LLMs with a contextual memory of prior choices did not stabilize their preferences. Adherence to MMTP and WST decreased under Memory-Full at both the mean and the model levels, and the strict MCT and HCT conditions remained violated under both conditions. Additionally, adherence in the 10 randomized orderings of the trial sequence varied significantly within the Memory-Full condition. The supplementary counterbalance consistency (Appendix B.1.1) further showed that LLMs failed to reproduce the same choice on counterbalanced pairs even when their earlier presentation was the immediately preceding prompt. None of these patterns is what a utility-following agent would produce. Such a system should remain stable regardless of the shape of its output distribution, should treat equivalent representations of the same probability as equivalent, and should maintain consistent preferences across trials.

When LLMs failed to maintain transitivity, violations were not randomly distributed across all possible cyclic preferences. Rather, they tended to concentrate in the direction previously documented in human decision-makers, with LLMs producing choice probabilities consistent with an intransitive lexicographic semi-order decision rule (Tversky, 1969). What is interesting to note is that rates of LLM transitivity violation is far higher for these stimuli sets than was observed for human respondents (Regenwetter et al., 2011, 2010; Davis-Stober et al., 2015; Cavagnaro and Davis-Stober, 2014).

It is important to clarify that our framework evaluates whether LLM outputs occupy a robust, structured, and consistent space of decisions, it does not reveal the processes by which those outputs are generated. The structural pattern we document is a claim about the structure of outputs, not about internal mechanisms. Extending the framework to process-level questions would require methods beyond transitivity testing, such as mechanistic interpretability or analysis of intermediate reasoning traces (Elhage et al., 2021; Olah et al., 2020).

While we illustrated our framework with simple binary gambles as choice alternatives, the framework itself could be readily applied to any set of choices. Future work should extend it to domains where LLMs are increasingly being deployed, such as medical triage, credit assessment, and moral trade-offs. Our environmental manipulations, such as memory manipulation, could be similarly applied and/or extended to other choice contexts.

Future work could consider evaluating LLMs using additional choice axioms derived from measurement theory and utility theory. Tests of monotonicity and the Thomsen conditions (e.g., Luce, 2000) could provide additional insight into the structure of LLM responses, specifically how risk interacts with outcomes. The current paper serves as a first step in a measurement oriented evaluation framework for LLMs.

## Figures and Tables

**Table 1:** Gamble Sets Used in the Experiment with Specific Probability and Monetary Value

Gamble Set	A		B		C		D		E	
	Prob	Val	Prob	Val	Prob	Val	Prob	Val	Prob	Val
Tversky 1	7/24	5.00	8/24	4.75	9/24	4.50	10/24	4.25	11/24	4.00
Tversky 2	8/24	5.00	10/24	4.75	12/24	4.50	14/24	4.25	16/24	4.00
Tversky 3	7/24	3.70	8/24	3.60	9/24	3.50	10/24	3.40	11/24	3.30
Davis-Stober 1	7/24	25.43	8/24	24.16	9/24	22.89	10/24	21.62	11/24	20.35
Davis-Stober 2	7/24	31.99	8/24	27.03	9/24	22.89	10/24	19.32	11/24	16.19

*Note*. Prob = Probability of Winning; Val = Monetary Value of Winning.

**Figure 6:**
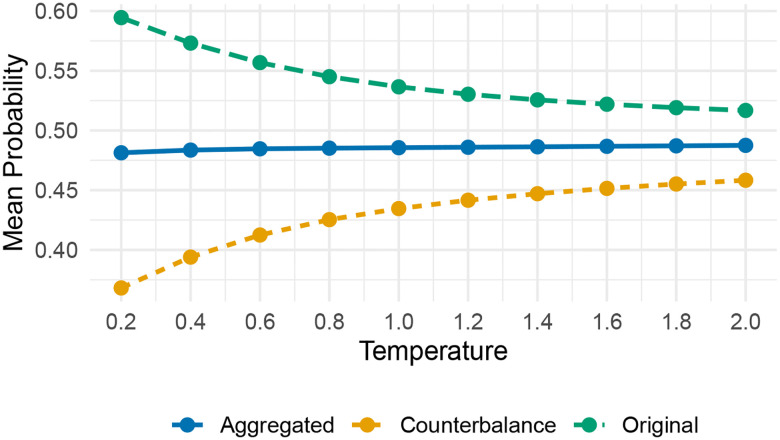
Mean choice probability across all 20 LLMs as a function of generation temperature for the Original, Counterbalance, and Aggregated conditions. The Original condition reflects the probability assigned to the first-presented gamble; the Counter-balance condition reflects the probability when presentation order is reversed. The Aggregated condition is the mean of the two.

**Figure 7:**
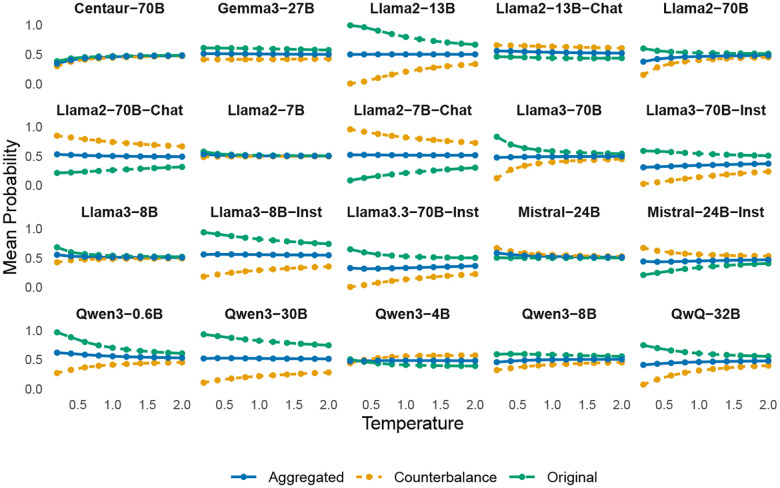
Mean choice probability as a function of generation temperature for the Original, Counterbalance, and Aggregated conditions, shown separately for each 20 LLMs.

**Figure 8:**
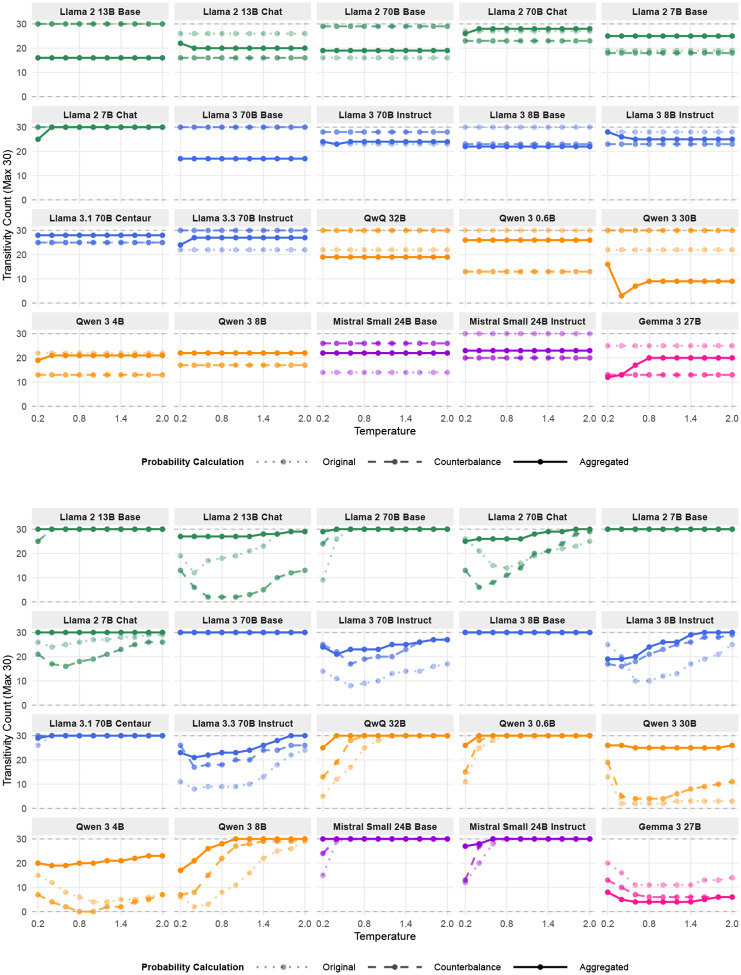
WST and MMTP adherence by model and temperature for the Original, Counterbalance, and Aggregated conditions (Top = WST, Bottom = MMTP).

**Figure 9:**
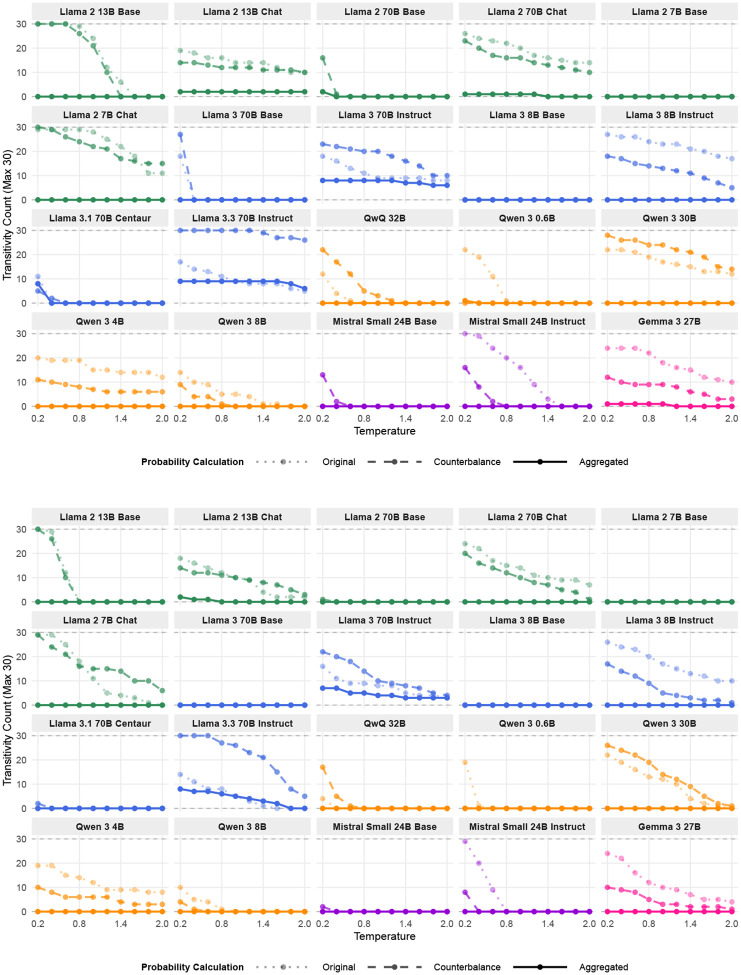
MCT and HCT adherence by model and temperature for the Original, Counterbalance, and Aggregated conditions.

**Figure 10:**
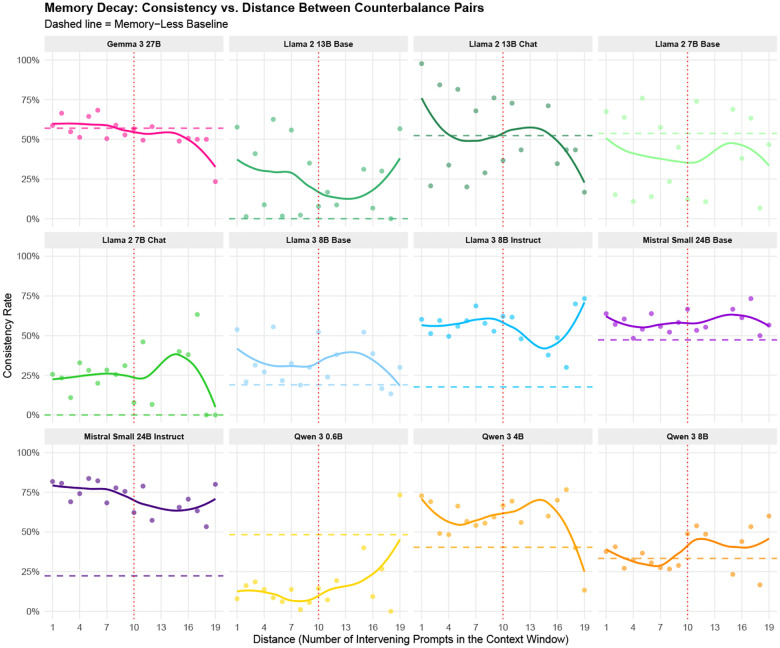
Consistency rate as a function of the distance between counterbalanced mirror pairs in the Memory-Full condition, shown separately for each of the 12 LLMs. X-axis indicates the number of intervening prompts between the two presentations of a mirror pair within the context window. The red dashed vertical line at distance 10 indicates the boundary of memory window. The dashed horizontal line in each panel represents the corresponding Memory-Less baseline consistency rate. Solid lines reflect the LOESS smoothing of the per-model consistency rates.

## Data Availability

The code required to reproduce all experiments and analyses is publicly available at https://github.com/stoberc-lab/llm_transitivity_gambles. The datasets analyzed in this study were generated by running the open-weight models listed in the Appendix at their specified Hugging Face revision IDs and can be regenerated deterministically from the available code.

## Footnotes

1 A comprehensive analysis quantifying the positional bias is detailed in Appendix B.1.

## A Appendix

### A.1 Models Evaluated

Table 2 lists the 20 LLMs evaluated in this study. The table is organized by developer, and Hugging Face revision IDs are provided to ensure exact reproducibility.

**Table 2:** LLMs Used for the Experiment by Developer, Name, Type, and Revision ID

Model	Name	Type	Revision ID
**Meta**			
Llama 2	Llama-2-7b-hf	Base	01c7f73d771dfac7d292323805ebc428287df4f9
	Llama-2-7b-chat-hf	Chat/Instruct	f5db02db724555f92da89c216ac04704f23d4590
	Llama-2-13b-hf	Base	5c31dfb671ce7cf2d7bb7c04375e44c55e815b1
	Llama-2-13b-chat-hf	Chat/Instruct	a2cb7a712bb6e5e736ca7f8cd89167f81a0b5bd8
	Llama-2-70b-hf	Base	3aba440b59558f995867ba6e1f58f21d0336b5bb
	Llama-2-70b-chat-hf	Chat/Instruct	e9149a12809580e860299585f8098ce973d1080
Llama 3	Meta-Llama-3-8B	Base	62bd457b6fe961a42a31306577e622c83876cb6
	Meta-Llama-3-8B-Instruct	Chat/Instruct	e1945c40cd546c78e4f1115ff4db032b271faeaa
	Meta-Llama-3-70B	Base	b4d08b7db494d88da3ac49adf25a6b9ac01ae338
	Meta-Llama-3-70B-Instruct	Chat/Instruct	7129260dd8548a80e1c0ace5f61c20324b472b31c
	Llama-3.1-Centaur-70B	Fine-Tuned	fde15959d0e34299b6abf7cb67ea00580fbdde71
	Llama-3.3-70B-Instruct	Chat/Instruct	6f6073b423013f6a7d4d9f39144961bfbfbc386b
**Alibaba**			
Qwen 3	Qwen3-0.6B-Chat	Chat/Instruct	e6de91484c29aa9480d55605af694f39b081c455
	Qwen3-4B-Chat	Chat/Instruct	531c80e289d6cff3a7cd8c0db8110231d23a6f7a
	Qwen3-8B-Chat	Chat/Instruct	9c925d64d72725edaf899c6cb9c377fd0709d9c5
	Qwen3-30B-A3B	Chat/Instruct	ae659febe817e4b3ebd7355f47792725801204c9
	QWQ3-32B-Reasoning	Fine-Tuned	976055f8c83f394f35dbd3ab09a285a984907bd0
**Mistral AI**			
Mistral	Mistral-Small-24B	Base	50a43a1f4488595def221f672346761c85ad90ea
	Mistral-Small-24B-Instruct	Chat/Instruct	20b2ed1c4e9af44b9ad125f79f713301e27737e2
**Google**			
Gemma 3	Gemma-3-27B-it	Chat/Instruct	005ad3404e59d6023443cb575daa05336842228a

### A.2 Stimuli and Prompts

#### A.2.1 Question Format Examples

Six question formats constructed by combination of three representations of monetary value(Numeric, Dollar Sign $, word “Dollars”) with two representations of winning probability (Fraction, Percentage). One example of each is provided below.

##### Numeric + Fraction

Gamble 1 can give 25.43 with a chance of 7/24. Gamble 2 can give 24.16 with a chance of 1/3. Which do you choose?

##### Numeric + Percentage

Gamble 1 can give 25.43 with a chance of 29.17%. Gamble 2 can give 24.16 with a chance of 33.33%. Which do you choose?

##### Dollar Sign + Fraction

Gamble 1 can give $25.43 with a chance of 7/24. Gamble 2 can give $24.16 with a chance of 1/3. Which do you choose?

##### Dollar Sign + Percentage

Gamble 1 can give $25.43 with a chance of 29.17%. Gamble 2 can give $24.16 with a chance of 33.33%. Which do you choose?

##### “Dollars” + Fraction

Gamble 1 can give 25.43 dollars with a chance of 7/24. Gamble 2 can give 24.16 dollars with a chance of 1/3. Which do you choose?

##### “Dollars” + Percentage

Gamble 1 can give 25.43 dollars with a chance of 29.17%. Gamble 2 can give 24.16 dollars with a chance of 33.33%. Which do you choose?

#### A.2.2 Memory-Less Prompt Templates

In the Memory-less condition, each trial was presented independently with no prior context. Instruct and Base models received different formats. Instruct prompts used the model’s appropriate chat template, while Base prompts used a plain text format.

##### Instruct model

<|begin_of_text|><|start_header_id|>system<|end_header_id|>

You have the choice of two gambles. Pick which one you would prefer.

<|eot_id|><|start_header_id|>user<|end_header_id|>

Gamble 1 can give 5.0 with a chance of 7/24. Gamble 2 can give 4.75 with a chance of 8/24. Which do you choose? <|eot_id|><|start_header_id|>assistant<|end_header_id|> I choose Gamble //

##### Base model

You have the choice of two gambles. Pick which one you would prefer.

Gamble 1 can give 25.43 with a chance of 29.17%. Gamble 2 can give 24.16 with a chance of 33.33%.

Which do you choose?

I choose Gamble

#### A.2.3 Memory-Full Prompt Templates

In the Memory-Full condition, each new trial was provided with the model’s prior choices within the gamble set. The examples below demonstrates two prior trials followed by the current trial. Up to 10 prior choices were given before each new trial.

##### Instruct model

<|system|>

You have the choice of two gambles. Pick which one you would prefer. </s>

<|user|>

Gamble 1 can give 21.62 with a chance of 10/24.

Gamble 2 can give 25.43 with a chance of 7/24.

Which do you choose ? </s>

<|assistant|>

I choose Gamble 2 </s>

<|user|>

Gamble 1 can give 24.16 with a chance of 8/24.

Gamble 2 can give 22.89 with a chance of 9/24.

Which do you choose ? </s>

<|assistant|>

I choose Gamble 2 </s>

<|user|>

Gamble 1 can give 22.89 with a chance of 9/24.

Gamble 2 can give 20.35 with a chance of 11/24.

Which do you choose?</s>

<|assistant|>

I choose Gamble

##### Base model

You have the choice of two gambles. Pick which one you would prefer.

Gamble 1 can give 21.62 with a chance of 10/24.

Gamble 2 can give 25.43 with a chance of 7/24.

Which do you choose ?

I choose Gamble 2

Gamble 1 can give 24.16 with a chance of 8/24.

Gamble 2 can give 22.89 with a chance of 9/24.

Which do you choose ?

I choose Gamble 2

Gamble 1 can give 22.89 with a chance of 9/24.

Gamble 2 can give 20.35 with a chance of 11/24.

Which do you choose ?

I choose Gamble

### A.3 Experimental Design Details

#### A.3.1 Memory-Full Presentation Ordering

To control for order effect in the Memory-Full condition, ten distinct presentation orderings of the 20 counterbalanced choice pairs were generated.

**Table 3:** The ten randomized presentation orderings used in the Memory-Full condition. Rows represents trial position within the sequence (1–20); columns represents the ordering. Each cell reports the counterbalanced pair presented at that trial, where **X_Y** denotes Gamble X first, Gamble Y second.

Trial	Order 1	Order 2	Order 3	Order 4	Order 5	Order 6	Order 7	Order 8	Order 9	Order 10
1	D_A	A_B	E_B	E_D	A_B	D_C	E_C	C_A	E_D	B_D
2	A_D	B_D	C_D	E_A	E_B	E_D	B_A	D_E	E_C	C_D
3	B_D	B_A	D_C	E_B	B_A	A_C	B_D	E_A	B_E	A_D
4	B_C	D_C	D_E	A_B	B_C	E_A	A_C	B_A	E_A	D_B
5	C_E	E_B	B_E	B_D	E_A	D_A	E_D	B_D	C_B	D_E
6	D_C	C_D	C_A	A_C	E_C	B_C	A_E	D_A	A_E	C_E
7	A_C	C_E	A_E	C_A	A_E	D_B	E_B	D_B	D_B	A_C
8	E_C	D_B	E_D	D_E	D_A	C_A	A_D	A_B	A_C	E_C
9	A_E	E_C	C_E	C_E	C_E	A_B	D_A	C_D	A_B	D_C
10	E_D	A_C	B_C	B_E	D_C	E_B	C_B	C_B	B_D	A_B
11	C_D	C_A	D_A	A_D	E_D	C_E	D_E	A_E	C_D	B_E
12	C_A	B_E	A_B	D_B	C_D	B_D	D_B	A_D	D_C	E_B
13	E_A	B_C	B_A	B_A	B_E	E_C	C_E	B_C	B_C	B_A
14	D_E	A_E	C_B	E_C	A_C	C_B	B_C	E_C	C_E	E_D
15	B_E	D_A	D_B	C_B	B_D	A_E	C_D	E_B	C_A	A_E
16	C_B	E_D	A_C	D_A	C_B	A_D	B_E	A_C	B_A	E_A
17	E_B	C_B	A_D	B_C	D_B	C_D	E_A	B_E	A_D	C_A
18	D_B	D_E	E_C	A_E	D_E	D_E	D_C	E_D	D_A	B_C
19	A_B	E_A	E_A	C_D	A_D	B_E	C_A	D_C	E_B	C_B
20	B_A	A_D	B_D	D_C	C_A	B_A	A_B	C_E	D_E	D_A

## B Supplementary Analyses

### B.1 Position Bias in Choice Probability

As described in Section 3.1, all transitivity analyses in the main text used Aggregated choice probabilities to control for positional bias. This section presents the full analysis of the bias and examines how it influences transitivity adherence when left uncorrected.

Figure 6 presents the mean choice probability across all 20 LLMs for the Original, Counterbalance, and Aggregated conditions as a function of generation temperature. Each point in the figure represents the average of all first-token probabilities assigned to the first-listed alternative, for each choice pair, across 20 LLMs, 5 gamble sets, and 6 question formats. For the Counterbalance condition, the probability was reversed (i.e., 1-P) so that all three conditions reflect the probability selecting the same gamble, allowing for a direct comparison of positional effect.

Across all temperature levels, a positional bias was observed, where the Original condition consistently exceeded 0.5, while the Counterbalance condition consistently fell below 0.5. The difference between the two was largest at temperature 0.2 (0.595 vs. 0.368) and smallest at temperature 2.0 (0.517 vs 0.458). This pattern indicates a systematic first-position bias in which models assign higher probability to whichever gamble occupied the first-presented option, regardless of its content. As temperature increased, both conditions gradually converged toward 0.5, which is expected due to the effect of temperature on the softmax distribution, where higher temperature flattens the output distribution. The Aggregated choice probability, computed as the mean of the Original and Counterbalance values, remained nearly flat across all temperatures, indicating that counterbalancing effectively mitigates the position bias.

While the grand mean reflects a general first-position bias, model-level analysis revealed considerable variation (see Appendix Figure 7). Most models favored the first-presented option, but a subset, including Llama 2 7B Chat, Llama 2 70B Chat, and Mistral Small 24B Instruct, consistently favored the second position. This reversal was particularly pronounced within the Llama 2 family, where base models favored the first-presented option, while their Chat counterparts favored the second. A separate group of models, including Centaur 70B, Llama 2 7B, Llama 3 8B, Mistral 24B, and Qwen 3 4B, produced choice probabilities near 0.5 across all temperatures, indicating persistent uncertainty between the two alternatives regardless of temperature.

To assess whether positional bias influenced transitivity adherence, the same analysis was conducted on the Original and Counterbalance probabilities as well. The results differed from the Aggregated analysis substantially (see Appendix Figures 8, and 9). For MMTP, Original and Counterbalance adherence was lower than Aggregated at low temperatures and increased toward the Aggregated level as temperature rose. This pattern may be due to the effect of temperature on choice probabilities: at low temperatures, the positional bias produces extreme probabilities that are more likely to violate the MMTP inequality $\left(P_{AB}+P_{BC}-P_{AC}\leq 1\right)$, while higher temperatures compress probabilities toward 0.5, making the inequality easier to satisfy. For MCT and HCT, the pattern was reversed. At temperature 0.2, the mean Original MCT count was 17.1 and the mean HCT count was 14.2, both higher than the near-zero Aggregated values. Both declined gradually with temperature. This collapse in adherence after aggregation may indicate that the strict transitivity observed in the uncorrected conditions was driven by positional bias inflating choice probabilities, rather than by the preferences of the LLMs themselves.

#### B.1.1 Memory Decay and Trial Consistency

This analysis examined whether contextual memory produced stable preferences across the context window, specifically, whether the model preferred the same gamble in both instances when a counterbalanced pair (e.g., AB and BA) was presented at two points in the sequence. To address this, we examined whether consistency between counterbalanced pairs varied based on the number of intervening prompts, which is referred to as “distance” separating the two presentations. A consistency rate was computed for each model at each distance, and compared against the Memory-Less baseline. Results across the 12 models are presented in Appendix Figure 10.

A standard prediction from the context-window literature is that LLMs attend most strongly to recent information in the context window, with consistency declining as the distance between two related events grows (Liu et al., 2024a; Laban et al., 2025). The observed pattern showed only a small decline of this kind. Across all 12 models, the mean consistency rate was 0.470 at near distances (1 to 5) and 0.417 at far distances (6 to 10). More striking was the level of consistency at short distances. Even at distance 1, where the earlier presentation of an order switched pair was the immediately preceding prompt in the context window, the mean consistency rate was 0.571, with large variations across models (range: 0.079 to 0.977). Combined with the small decline across distance, this pattern suggests that LLMs were failing to commit to a stable choice regardless of how recent the prior choice was.

Mean consistency varied widely across the 12 models, ranging from 0.17 for **Qwen 3 0.6B** to 0.72 for **Mistral Small 24B Instruct**. Five models maintained mean consistency above 0.55 (Mistral Small 24B Instruct and Base, Qwen 3 4B, Llama 3 8B Instruct, and Gemma 3 27B), three remained below 0.30 (Qwen 3 0.6B, Llama 2 13B Base, and Llama 2 7B Chat), and the remaining four fell between. Some models reproduced their preference across counterbalanced presentations more reliably than others, even within the same memory window.

These results suggest that contextual memory does not function as a preference stabilizer. Even when prior choices were within the memory window, most models did not reliably reproduce the same underlying preference across counterbalanced presentations. This pattern aligns with the main results reported in Section 3.3, where contextual memory reduced MMTP and WST adherence rather than stabilizing it. In addition, the within-model variability across the 10 randomized orderings indicated that the structural consistency of preferences depended on the sequence in which prior choices were encountered. Together, the two analyses point toward a common conclusion that the contextual record of prior choices does not produce consistent preferences across the context window.
